# Differential Diagnosis in Patients Presenting With Peripheral Lymphadenopathy: Tuberculosis, Sarcoidosis, or Malignancy

**DOI:** 10.7759/cureus.93919

**Published:** 2025-10-06

**Authors:** Esma Tuğba Canlı, Merve Erçelik, Önder Öztürk, Hasan Yasan, İbrahim Metin Çiriş

**Affiliations:** 1 Department of Chest Disease, Süleyman Demirel University Faculty of Medicine, Isparta, TUR; 2 Department of Otolaryngology - Head and Neck Surgery, Süleyman Demirel University Faculty of Medicine, Isparta, TUR; 3 Department of Pathology, Süleyman Demirel University Faculty of Medicine, Isparta, TUR

**Keywords:** active pulmonary tuberculosis, extrapulmonary tuberculosis (eptb), lung cancer, microbiological evaluation, peripheral lymphadenopathy, pulmonary sarcoidosis

## Abstract

Differential diagnosis of peripheral lymphadenopathies is important in terms of not missing infections or non-infectious diseases and early diagnosis of malignancies. Preliminary diagnoses of tuberculosis, sarcoidosis, and malignancy were considered for two clinically similar patients, a 33-year-old man and a 41-year-old woman, who presented to our clinic with neck swelling. The male patient's exposure to tuberculosis and the chest x-ray brought us closer to the diagnosis of tuberculosis reactivation. The female patient's multiple peripheral lymphadenopathies and metastasis-suspicious lesions seen on positron emission tomography increased our suspicion of malignancy. However, in the final diagnostic process, the female patient was diagnosed with tuberculous lymphadenitis, and the male patient was diagnosed with sarcoidosis. We presented these two cases to show that it isn't always easy to distinguish tuberculosis and sarcoidosis with clinical, radiological, laboratory, and histopathological findings, and to draw attention to the importance of microbiological evaluation.

## Introduction

The human body contains approximately 600 lymph nodes. Peripheral lymph nodes are located in the subcutaneous tissue and can be palpated when pathological processes cause them to enlarge. Lymphadenopathy (LAP) refers to conditions in which lymph nodes become abnormal in size, consistency, or number [1,2].

The differential diagnosis of peripheral LAP is crucial for the early detection and management of infectious and non-infectious diseases, and for not overlooking malignancies. LAP may result from a variety of causes, including tuberculosis (TB), sarcoidosis, and malignancies, all of which can present with overlapping clinical features. Initial evaluation typically includes a detailed clinical assessment and radiological examination of the head and neck. When findings suggest an inflammatory origin, further investigations such as microbiological and serological tests, as well as fine-needle aspiration biopsy or excisional biopsy, are required to establish a definitive diagnosis [1-3].

In this report, we present two patients from Türkiye with similar clinical complaints and a preliminary suspicion of TB, sarcoidosis, or malignancy, aiming to compare the diagnostic processes. Considering the global burden of TB and the relatively rare but increasingly recognized incidence of sarcoidosis in Türkiye, these cases highlight the challenges in differentiating between these conditions.

## Case presentation

Case 1

A 33-year-old male patient presented with a swelling in his neck that he noticed two days ago. He also had symptoms of sputum, night sweats, and cough for five days. It was learned that the patient didn’t smoke and had no other disease, but his father had a history of TB. The patient was working as a teacher.

On physical examination of the patient, whose vital signs were normal, there was a firm, mobile lymphadenopathy in the right submandibular area measuring approximately 2 cm in diameter. The lymph node was not matted, and the overlying skin appeared normal. Crackles were heard in the lung apices on auscultation. Ophthalmologic examination was also performed and revealed no ocular involvement.

**Figure 1:**
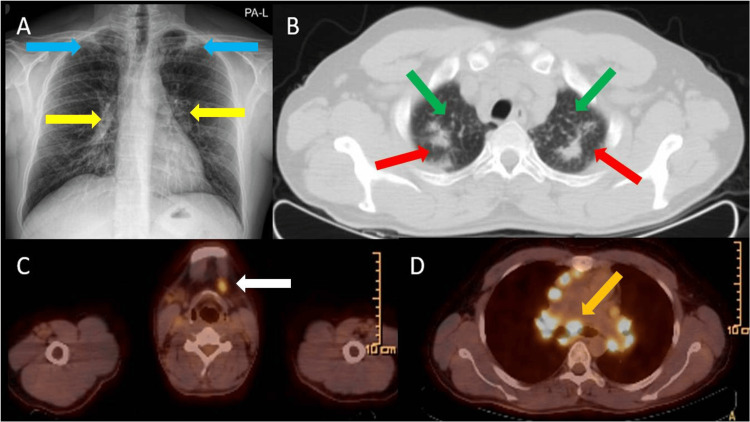
Imaging findings in Case 1 (A) Chest X-ray shows infiltrate areas in the upper regions (blue arrows) and bilateral hilar fullness (yellow arrows); (B) CT shows, at the apex level of both lungs, areas of post-tuberculosis fibrotic changes (red arrows) and millimetric centrilobular scattered nodules in the upper lobes of both lungs (green arrows); (C) PET/CT shows hypermetabolic lymph nodes in the deep cervical region bilaterally, the largest on the left being 1.5 cm (SUV_max_: 4.54) (white arrow); (D) PET/CT shows many hypermetabolic lymph nodes; the largest in the subcarinal region is 4 cm (SUV_max_: 13.1) (orange arrow). SUV_max_: maximum standardized uptake value

A chest x-ray was taken (Figure 1A). The patient's thorax computed tomography (CT) showed lymph nodes in the mediastinum and bilateral hilar regions, the largest of which was 31x19 mm, which could be compatible with an enlarged granulomatous lesion. Parenchymal changes were evaluated as post-TB fibrotic changes, predominantly affecting both upper lobes (Figure 1B).

The patient's blood values were normal except that the angiotensin-converting enzyme (ACE) level was 103 U/L (normal range 12-82 U/L). Tumor markers, including carcinoembryonic antigen (CEA), cancer antigen 125 (CA-125), and cancer antigen 19-9 (CA 19-9), were within normal limits. With the suspicion of TB, sputum culture, acid-fast bacilli (AFB), and tuberculin skin test (PPD) were performed. The PPD was positive, but AFB results were negative, and no growth was observed in the sputum culture.

Due to suspicion of malignancy, a positron emission tomography (PET)/CT scan was performed, which revealed multiple hypermetabolic lymph nodes in the head and neck region, thorax, abdomen, and inguinal area (Figures 1C, 1D). The patient declined bronchoscopy and endobronchial ultrasound (EBUS) procedures and was referred to the ear, nose, and throat clinic for a biopsy.

**Figure 2:**
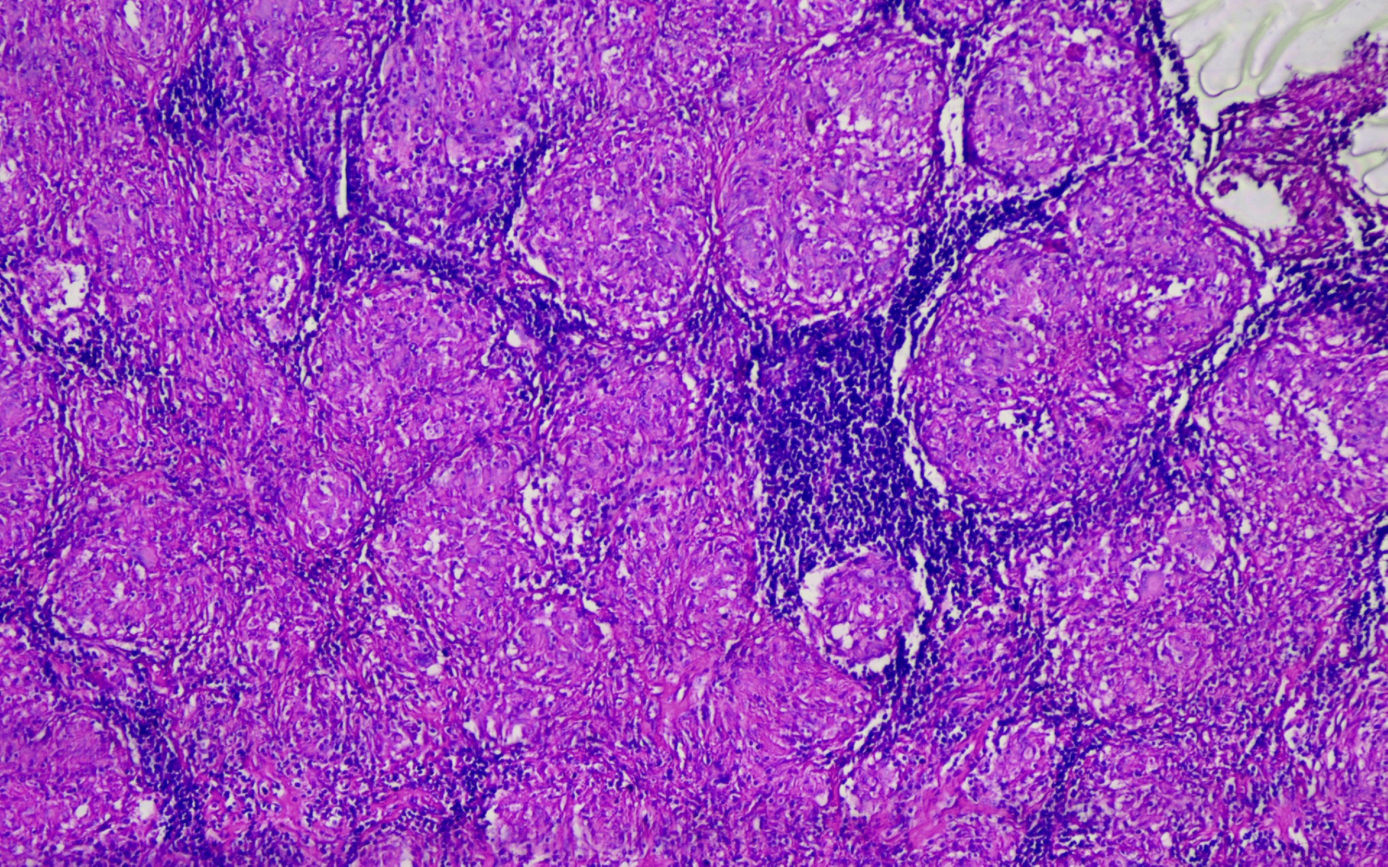
Histopathological findings in Case 1 Non-caseating granulomas are seen disrupting the normal structure of the lymph node. Multinucleated giant cells are visible. 200x, H&E.

The excisional biopsy taken from the left cervical region resulted in non-caseating granulomatous lymphadenitis, and there was no growth in tissue culture (Figure 2).

The patient was diagnosed with Stage 1 sarcoidosis. Given the absence of progressive symptoms or significant organ involvement, the patient was followed up without systemic corticosteroid therapy. The patient was monitored every three months for 24 months, with no clinical or radiological progression observed.

Case 2

A 41-year-old female patient was admitted with complaints of neck swelling, joint pain, palpitations, weight loss, fatigue, night sweats, cough, and white sputum for a week. She had never smoked, didn't have any chronic diseases, and was working as a farmer.

**Figure 3:**
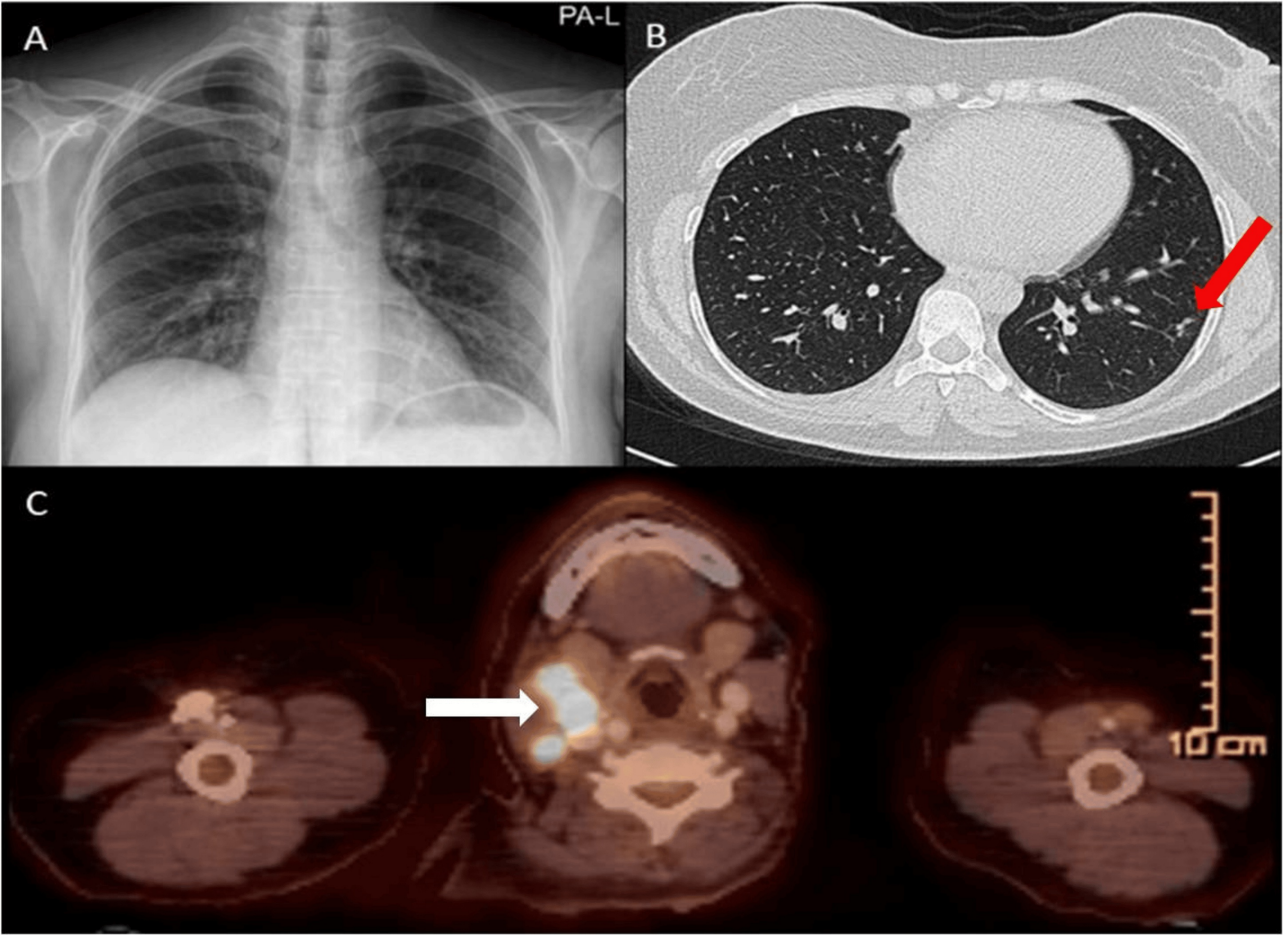
Imaging findings in Case 2 (A) No pathological area observed in chest X-ray; (B) HRCT shows several millimetric nodules in the peripheral area (red arrow); (C) PET/CT shows multiple hypermetabolic lymph nodes; most notably 16 mm (SUV_max_: 14.27) in the right level IIa (white arrow). HRCT: high-resolution computed tomography; SUV_max_: maximum standardized uptake value

On physical examination, multiple bilateral submandibular and cervical lymphadenopathies were palpated, ranging between 1.5 to 2 cm in size. The nodes were soft, discrete, and mobile, with no signs of matting or tenderness. Ophthalmologic examination showed normal findings, with no evidence of ocular involvement. X-ray (Figure 3A) and high-resolution CT (HRCT) of the lungs (Figure 3B) were done.

In the laboratory work-up, C-reactive protein (CRP) was 12.6 mg/L, and erythrocyte sedimentation rate (ESR) was 20 mm/hour. Tumor markers, including CEA, CA-125, and CA 19-9, were within normal limits. ACE level was measured at 44 U/L. The PPD was positive, but the sputum culture and AFB results were negative.

To investigate the malignancy, a PET/CT scan was performed, which revealed multiple hypermetabolic lymph nodes in the head and neck region with suspected metastasis (Figure 3C). The patient was referred to the ear, nose, and throat clinic for a biopsy, but she declined the bronchoscopy and EBUS.

**Figure 4:**
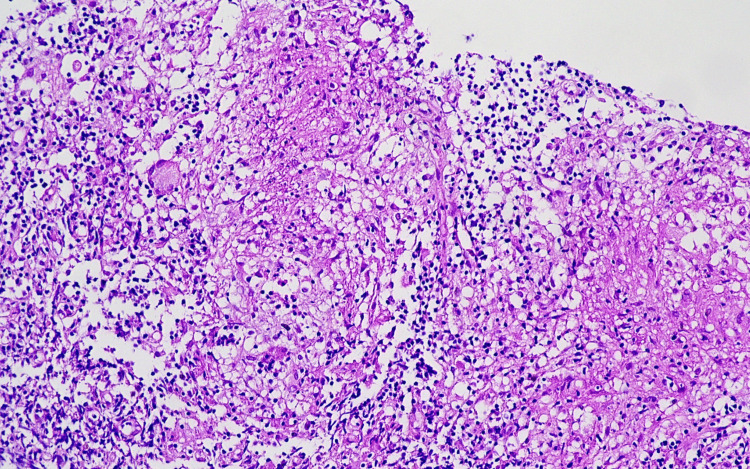
Histopathological findings in Case 2 Abortive granulomas in a trucut biopsy of the lymph node. A multinucleated giant cell is observed on the left. 200x, H&E.

The excisional biopsy result taken from the right submandibular region also resulted in lymphoid tissue accompanied by non-caseating granulomas. However, the patient's tissue culture resulted in *Mycobacterium tuberculosis* Complex (Figure 4). Histopathologically, a diagnosis of grade II (non-caseating epithelioid granulomatous reaction) tuberculous lymphadenitis was made.

Based on clinical findings and imaging, the patient was classified as having Stage 2 (uncomplicated) tuberculous lymphadenitis, as there were no signs of abscess formation, fistula, or sinus tract, and the disease was confined to peripheral lymph nodes. She was started on standard anti-tuberculosis therapy in accordance with the American Thoracic Society (ATS) guidelines, with an intensive phase of two months using isoniazid, rifampin, pyrazinamide, and ethambutol (HRZE), followed by a continuation phase of four months with isoniazid and rifampin (RH). Clinical improvement was noted after two months, including regression in lymph node size and resolution of systemic symptoms. A complete cure was achieved after the six-month standard anti-tuberculosis therapy. The patient was monitored monthly during treatment and subsequently every six months for a total of 24 months, with complete clinical and radiological resolution observed and no recurrence reported.

Comparison

For ease of comparison, Table 1 provides a side-by-side summary of the key clinical, laboratory, imaging, histopathological, and microbiological features of the two cases, highlighting the diagnostic decision-making process.

**Table 1:** Summary and comparison of the distinguishing features of both cases ACE: angiotensin-converting enzyme; CT: computed tomography; FDG: fluorodeoxyglucose; LAD: lymphadenopathy; PCR: polymerase chain reaction; PET: positron emission tomography; TB: tuberculosis

Parameter	Case 1 – Sarcoidosis	Case 2 – Tuberculous Lymphadenitis
Age, Sex	33 years, Male	42 years, Female
Main Symptoms	Fatigue, night sweats, productive cough, sputum	Fatigue, low-grade fever, rapidly enlarging lymph nodes
Family History	Father with history of pulmonary TB	No remarkable family history
Lymphadenopathy	Bilateral hilar, mediastinal, cervical	Cervical and mediastinal
Laboratory Findings	Elevated ACE, normal inflammatory markers, normal tumor markers	Normal ACE, mildly elevated inflammatory markers, normal tumor markers
Imaging (X-ray, CT, PET)	Centrilobular nodules, bilateral hilar LAD, moderate FDG uptake	Multiple hypermetabolic lymph nodes, moderate FDG uptake, no lung lesions
Histopathology (excisional lymph node biopsy)	Non-caseating granulomas	Non-caseating granulomas
Microbiology (tissue culture)	All cultures and PCR negative for TB	Culture positive for Mycobacterium tuberculosis
Final Diagnosis	Sarcoidosis	Tuberculous lymphadenitis
Timeline	Gradual onset, symptoms resolved spontaneously without anti-TB therapy	Progressive symptoms until anti-TB therapy initiated
Response to Therapy	Follow up without systemic corticosteroid therapy	Resolution after anti-TB therapy

## Discussion

TB and sarcoidosis are chronic, multi-systemic, granulomatous diseases that have similar pulmonary and extra-pulmonary manifestations. Since both diseases have common clinical, radiological, and histological features, differential diagnosis is not always easy [4]. Especially in countries with a high burden of TB, differential diagnosis from sarcoidosis is difficult clinically [5]. Our study highlights the importance of a comprehensive, multimodal approach combining clinical findings, imaging, pathological, and microbiological testing.

Systemic symptoms such as fever, night sweats, fatigue, and weight loss are commonly observed in both sarcoidosis and TB lymphadenitis; however, as these two diseases significantly overlap, distinguishing them based solely on clinical symptoms is impractical, and any differences should be considered only as a reference rather than a definitive means of differentiation. TB often presents with more pronounced infectious symptoms, including productive cough and purulent sputum [6], as in our first case. In contrast, sarcoidosis typically manifests with more subtle constitutional symptoms such as dry cough, fatigue, and occasionally arthralgia or erythema nodosum [7].

In Case 1, the presence of purulent sputum, chronic cough, and constitutional symptoms raised a strong suspicion of pulmonary TB. However, microbiological investigations, including AFB staining and culture, were negative, and the patient's symptoms gradually resolved without anti-TB treatment during follow-up, further supporting the diagnosis of sarcoidosis. Case 2, on the other hand, presented with similar constitutional symptoms but also had rapidly enlarging lymphadenopathy. Despite initial suspicion of malignancy, culture confirmed TB lymphadenitis. These contrasting presentations reflect the clinical ambiguity often encountered in differentiating the two entities.

Although elevated ACE levels are known to support the diagnosis of sarcoidosis, their diagnostic utility is limited by variable sensitivity and specificity [8]. Laboratory findings provided supportive, but not definitive, clues in differentiating between the two conditions. In Case 1, ACE was elevated while all other routine blood tests were within normal ranges; tumor markers were also normal, helping to exclude malignancy. In Case 2, inflammatory markers were mildly elevated, while the ACE level was within normal limits, and tumor markers were again unremarkable. Although none of these laboratory findings are pathognomonic, when interpreted alongside radiologic and histopathologic features, they contributed meaningfully to the diagnostic reasoning in both patients.

Tuberculin skin testing (PPD) and interferon-gamma release assays (IGRAs) are frequently used to support TB diagnosis, but both have limitations. PPD has reduced specificity in Bacillus Calmette-Guérin(BCG)-vaccinated populations and in regions with prevalent environmental mycobacteria. IGRA offers higher specificity (~95%) but variable sensitivity, particularly in extrapulmonary or latent TB [9]. Both patients in this report had positive PPD results, which were considered likely related to their residence in a TB-endemic region such as Türkiye. In our setting, IGRA was not available, which represents one of the limitations of our study. However, given the diagnostic limitations of both PPD and IGRA, and the absence of microbiological confirmation, these findings were not sufficient to establish a definitive diagnosis.

Imaging plays a crucial role in differential diagnosis, though findings may be nonspecific. In TB, reactivation typically manifests as centrilobular nodules, tree-in-bud opacities, and fibronodular infiltrates predominantly in the upper lobes [10]. In contrast, sarcoidosis commonly demonstrates bilateral hilar and mediastinal lymphadenopathy, perilymphatic nodules, and, in advanced stages, parenchymal fibrosis [11]. In Case 1, chest X-ray demonstrated bilateral hilar lymphadenopathy, while CT imaging revealed centrilobular nodules and fibrotic changes in the upper lobes. The parenchymal abnormalities were interpreted as post-TB fibrotic changes; however, the presence of bilateral hilar lymphadenopathy, in the absence of microbiological evidence of active infection, supported the diagnosis of sarcoidosis. In Case 2, the absence of significant parenchymal involvement but the presence of progressively enlarging peripheral lymph nodes necessitated further investigations to clarify the underlying pathology. Overall, these findings highlight that both chest X-ray and CT can suggest possible differential diagnoses, yet often pose diagnostic challenges without microbiological or histopathological confirmation.

It is known that FDG uptake seen in PET/CT is not disease specific and can be increased in both benign and malignant lymphoproliferative processes [12]. In Case 1, PET/CT showed hypermetabolic lymph nodes in the bilateral deep cervical region and additional hypermetabolic nodes in the mediastinal region. Although such FDG-avid lymphadenopathy can raise suspicion for malignancy, it is also well-documented in both TB and sarcoidosis. In Case 2, PET/CT demonstrated multiple hypermetabolic lymph nodes without pulmonary involvement, a pattern that can be seen in both sarcoidosis and extrapulmonary TB. These findings underscore the limited specificity of FDG uptake in distinguishing between granulomatous and malignant etiologies [13,14]. Thus, while PET/CT was valuable in lesion detection, it did not establish etiology without histopathological and microbiological correlation.

Histopathological analysis remains essential in evaluating granulomatous diseases. Granulomatous inflammation is a hallmark of both sarcoidosis and TB, yet they exhibit key histopathological differences. While non-caseating granulomas are characteristic of sarcoidosis, TB often presents with caseous necrosis. However, this distinction is not absolute. It is well-documented that up to 30% of TB lymphadenitis cases may demonstrate non-caseating granulomas, particularly in immunocompetent individuals [15]. As seen in our cases, both biopsies revealed non-caseating granulomas. The presence of multinucleated giant cells in both cases further highlights the shared immune-mediated nature of granulomatous inflammation in these diseases. In Case 1, despite clinical and radiological suspicion, all microbiological tests were negative, supporting a diagnosis of sarcoidosis. In Case 2, culture of biopsy material confirmed TB. Therefore, histopathology alone is insufficient for definitive diagnosis, and microbiological confirmation is indispensable [16]. These findings emphasize the importance of tissue diagnosis in combination with microbiological studies.

As extra-pulmonary manifestations, ocular involvement may occur in both TB and sarcoidosis, adding further complexity to the differential diagnosis. Ocular TB most commonly presents as chronic granulomatous uveitis, choroiditis, or retinal vasculitis, and can lead to significant visual morbidity if untreated [17]. Similarly, sarcoidosis is one of the leading causes of non-infectious uveitis worldwide and may present with anterior, intermediate, or posterior uveitis, keratoconjunctivitis sicca, or retinal periphlebitis [7]. In our cases, ophthalmologic examinations were within normal limits, and no ocular involvement was detected. Although the presence of ocular manifestations is not disease-specific, their recognition is important because ophthalmologic evaluation may provide additional diagnostic clues in patients presenting with peripheral lymphadenopathy and suspected granulomatous disease.

While sarcoidosis and TB are distinct granulomatous diseases, rare cases of their coexistence or sequential occurrence have been documented, particularly in TB-endemic regions. Kaur et al. described a patient with concurrent, microbiologically confirmed pulmonary TB and sarcoidosis, underscoring the diagnostic complexity of such presentations [18]. In another case, sarcoidosis was diagnosed during TB treatment, possibly reflecting immune reconstitution or the unmasking of a latent granulomatous process [19]. Furthermore, van Enschot and van Balkom suggested that sarcoidosis may develop as a post-infectious immunological response following TB infection, raising the question of whether sarcoidosis is, in selected cases, a consequence rather than a coincidence [20]. In light of these reports, our first case raises suspicion of a potential association between prior mycobacterial infection and subsequent sarcoidosis. The second case is currently under follow-up for the same reason. To draw more comprehensive conclusions on this topic, further multicenter studies involving larger cohorts are needed. In this report, all diagnoses were supported by both microbiological and histopathological findings, emphasizing the importance of a meticulous differential diagnosis-particularly in TB-endemic settings.

## Conclusions

These two cases illustrate the diagnostic challenges in distinguishing TB from sarcoidosis, especially in regions where TB is endemic. Despite overlapping clinical, radiological, and histopathological features, accurate diagnosis requires microbiological confirmation. Clinicians should consider both diseases in the differential diagnosis of peripheral lymphadenopathy and integrate all diagnostic modalities to avoid misdiagnosis.

## References

[1] Ferrer R (1998). Lymphadenopathy: differential diagnosis and evaluation. Am Fam Physician.

[2] Mohseni S, Shojaiefard A, Khorgami Z (2014). Peripheral lymphadenopathy: approach and diagnostic tools. Iran J Med Sci.

[3] Freeman AM, Matto P (2023). Lymphadenopathy. StatPearls [Internet].

[4] Agrawal R, Kee AR, Ang L (2016). Tuberculosis or sarcoidosis: opposite ends of the same disease spectrum?. Tuberculosis (Edinb).

[5] Babu K (2013). Sarcoidosis in tuberculosis-endemic regions: India. J Ophthalmic Inflamm Infect.

[6] Polesky A, Grove W, Bhatia G (2005). Peripheral tuberculous lymphadenitis: epidemiology, diagnosis, treatment, and outcome. Medicine (Baltimore).

[7] Judson MA (2015). The clinical features of sarcoidosis: a comprehensive review. Clin Rev Allergy Immunol.

[8] Hu X, Zou L, Wang S, Zeng T, Li P, Shen Y, Chen L (2022). Performance of serum angiotensin-converting enzyme in diagnosing sarcoidosis and predicting the active status of sarcoidosis: a meta-analysis. Biomolecules.

[9] Lewinsohn DM, Leonard MK, LoBue PA (2017). Official American Thoracic Society/Infectious Diseases Society of America/Centers for Disease Control and Prevention clinical practice guidelines: diagnosis of tuberculosis in adults and children. Clin Infect Dis.

[10] Alshoabi SA, Almas KM, Aldofri SA (2022). The diagnostic deceiver: radiological pictorial review of tuberculosis. Diagnostics (Basel).

[11] Criado E, Sánchez M, Ramírez J, Arguis P, de Caralt TM, Perea RJ, Xaubet A (2010). Pulmonary sarcoidosis: typical and atypical manifestations at high-resolution CT with pathologic correlation. Radiographics.

[12] Kamboj S, Goel MM, Tandon P (1994). Correlative study of histopathology and bacteriology in patients of tubercular lymphadenitis. Indian J Chest Dis Allied Sci.

[13] Maturu VN, Agarwal R, Aggarwal AN, Mittal BR, Bal A, Gupta N, Gupta D (2014). Dual-time point whole-body 18F-fluorodeoxyglucose PET/CT imaging in undiagnosed mediastinal lymphadenopathy: a prospective study of 117 patients with sarcoidosis and TB. Chest.

[14] Cengiz A, Aydın F, Sipahi M (2018). The role of F-18 FDG PET/CT in differentiating benign from malignant pulmonary masses and accompanying lymph nodes. Tuberk Toraks.

[15] Mukhopadhyay S, Gal AA (2010). Granulomatous lung disease: an approach to the differential diagnosis. Arch Pathol Lab Med.

[16] Ahmed HG, Nassar AS, Ginawi I (2011). Screening for tuberculosis and its histological pattern in patients with enlarged lymph node. Patholog Res Int.

[17] Konana VK, Babu K (2025). Current concepts in the diagnosis of ocular tuberculosis: a narrative review. Taiwan J Ophthalmol.

[18] Kaur H, Singh D, Pandhi N (2021). Co-existence of pulmonary tuberculosis with sarcoidosis. Int J Mycobacteriol.

[19] Cho HS, Kim SJ, Yoo JY (2021). Sarcoidosis during treatment of pulmonary tuberculosis: a rare case report and review of the literature. J Int Med Res.

[20] van Enschot JW, van Balkom RH (2013). Sarcoidosis following Mycobacterium tuberculosis infection: coincidence or consequence. Respir Med Case Rep.

